# Ten simple rules for postdoctoral mums to stay competitive in academia

**DOI:** 10.1371/journal.pcbi.1014145

**Published:** 2026-04-08

**Authors:** Belén Fadrique, Selene Báez

**Affiliations:** 1 School of Environmental Sciences, University of Liverpool, Liverpool, United Kingdom; 2 Departamento de Biología, Escuela Politécnica Nacional del Ecuador, Quito, Ecuador; 3 MODEMAT Foundation for Mathematical Modeling and Education, Quito, Ecuador; Carnegie Mellon University, UNITED STATES OF AMERICA

## Abstract

In academia, the intersection of the postdoctoral stage, usually highly unstable and decisive to secure a permanent position, and motherhood, is the most prominent culprit of the well-known problem of the decreasing number of female researchers in senior academic positions. The loss of postdoctoral women from the academic path represents an unsustainable loss of talent, leading to unbalanced academic institutions where this phenomenon eventually gets perpetuated. The motherhood challenges for postdoctoral women begin from the moment they plan on getting pregnant and continue well after reincorporation to work after maternity leave. Here, we provide 10 actionable rules for these postdoctoral women approaching motherhood to increase their chances of remaining in the academic career. These rules will help postdoctoral women prepare for the challenge of becoming a mother while working towards their long-term academic goals, and establish a successful relationship with their supervisors and collaborators under the new circumstances. These rules should be complemented by the general effort from colleagues, supervisors, institutions, and academia as a whole, to create a more supportive working environment. It is in the utmost interest of the academic community to improve the retention of postdoctoral mums and promote their progression to more senior positions.

## Introduction

Despite equal suitability and ambition, fewer women get to senior academic positions than their male counterparts [1–3]. This phenomenon has been referred to as the leaky pipeline, illustrating the constant “leakage” of women out of the academic career path [4]. There is some discussion as to whether this expression perpetuates a false idea of women passively slipping away from the system, as opposed to the more realistic situation where women are forced out of the system or expected to jump over an obstacle course [5]. Research shows that the single most important moment where women are forced out of the academic career is during their postdoc stage, where, in many cases, the combination of new maternity limitations and duties, and the need for long-term stability makes the progression of their academic career at a minimum, very challenging, at a maximum, unfeasible [6–8].

The postdoc stage is the moment in most researchers’ careers where they are expected to become independent and produce at the highest level in order to secure a permanent position [9]. For women, whether through pregnancy or adoption, having kids at this stage introduces a whole new set of priorities and challenges, which can/does affect their research output [6,10–12]. Even from the moment when women are considering starting a family, all travelling commitments (conferences, field work, secondments…) have to be reconsidered, and potentially cancelled [13]. This stage can be short or it can be incredibly long, depending on personal circumstances. During pregnancy, together with multiple medical appointments, there are different phases of physical distress, from daily vomiting to sciatica, which can be more or less bearable, affecting as much the number of hours one can be sitting in front of a computer, as the hours one can stand up in a lab. In addition, travelling during some of the pregnancy months or performing strenuous work, such as what is sometimes required during fieldwork, is unadvised. However, it is after birth that the real struggle begins. Maternity leave is an essential time to allow the mother to recover physically and mentally from labour and to look after and bond with the newborn baby. It is an equally important time for adoptive mothers to get to know their new child, bond, and develop a new family routine. For many mums, this time, whether cherished or not, comes at a cost, as the rest of the academic world is still frantically working, creating new collaborations, publishing papers, presenting at conferences, collecting data, recruiting students, speaking with the press, and submitting grants. Unless specific measures are taken, mums on maternity leave miss out on a lot of these activities. And finally, when mums return to work, they often learn about missed opportunities, they realise they have limited time in their contract, or they struggle with the new time constraints and the limitations to travelling, or working long-days, late-nights or weekends as they could resort to before [14]. This potential sudden decrease in competitiveness, together with the need to construct a support system that helps fulfil the new emotional and material/logistic needs of the family, may push postdoc mums to search for employment opportunities outside of academia [15,16].

As an academic society, we need to stop this constant loss of researchers [17]. By losing this generational group of women, we not only lose their scientific output, but also their skills gained through the experience of motherhood (e.g., multitasking, negotiation, troubleshooting, planning) [18–20], which are underemphasised but key to developing a more productive, diverse, balanced, and representative atmosphere at all academic levels [21]. The inclusion of mums in academia, and their mentorship and role-modelling is particularly important for recruiting and retaining the next generation of female academics, who could then infer that combining academia and motherhood is possible [7,22,23].

Unfortunately, the fate of postdoctoral women who have kids is not completely under their control. Some of the most important policies and actions that determine the future of these women depend on the country policies, with large variation in length of maternity leave (pre and post birth), percentage of salary paid, requirements to access the support, and partner rights/sharing conditions varying widely between countries. The most extreme examples could be the US, with a zero-week paid maternity leave policy, and Bulgaria, which offers 58.6 weeks at 90% salary [24]. Countries also differ in the minimum requirements that institutions must meet regarding benefits and provisions for working women entering a maternity period, which can be supplemented further by institutional policies, for example, by regulating equal-recruitment opportunities or allowing for flexible and/or remote work schedules.

**Fig 1:**
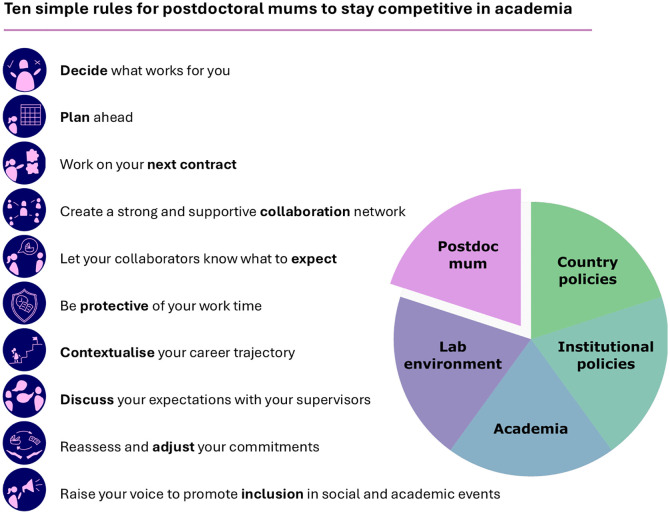
Infographic showing the proposed 10 rules for postdoctoral mums to stay competitive in academia, placed within the context of other academic-related actors that would determine their retention in the academic path.

Even with mum-friendly country-level policies, individual institutions, departments, and labs have different cultures that can be more or less supportive to these academics. There is a lot that supervisors and collaborators can do to support postdoctoral mums, for example, including them in developing projects or creating a mum-friendly lab (see Ten simple rules for a mum-friendly academia [25]). Although it is not a simple issue, we present here 10 actions (Fig 1) to be taken by the postdoctoral women who are recent mums or expecting to be on maternity leave and want to stay in academia, to help them increase their chances of remaining in this path. These are measures that women can take to minimise the impact of having kids on their careers, while still enjoying this new phase, which is crucial to retain them in academia. These rules fill the specific gap to support postdoctoral mums within the more general framework of creating a more inclusive and mum-friendly academia [25,26]. It is critical to realise that these rules alone, without a supportive environment, will probably not be enough to increase women’s retention in academia. Therefore, it is the job of everyone in the academic community to create this environment. It is also important to recognise that, depending on the lab/department culture, some of the rules may be better to skip. For example, if your supervisors are not allies on the cause, working on the other rules but telling them as late as possible (rule 8) may be more appropriate.

## 10 Rules

### 1. Decide what works for you

When it comes to maternity leave, some mums decide to take the minimum allowed and others use up all their allowance; some mums really enjoy disconnecting from work, while others prefer to keep in touch. When returning to work, some mums make use of full-day childcare while others reduce their working hours. Whatever your situation and your preference is, do not feel guilty about it. From now on, it is on you to find the right balance that works for you, your family and your job and to make active decisions about it. Avoid being carried away by circumstances or social pressure. Frustration and regret of how maternity worked out may push you to decide to change to a more family-friendly career [15].

### 2. Plan ahead

Planning is everything. There are two sections that need to be planned as soon as you know when you will be on leave: your goals before your maternity leave, and your return after your leave. Your pre-leave goals need to be reasonable, tactical, and strategic. Any process that needs time or that can be in someone else’s hands while you are on leave is a priority. For example, manuscript submission (it usually takes a few months to get back to you - and you can then ask for an extension to reply to the corrections), collaborators’ feedback on grants, permit approval, etc. If you have students or technicians, you could delegate some tasks – think about it as an opportunity for them to excel at something new.

Your post-leave goals need to be detailed. After a few months of being disconnected from work, returning to a messy situation can exacerbate the feeling of a lack of belonging. Before your leave, write a private report with every task you are doing, what the status is, and a list of actionable items for your return. This should give you some structure and make your first weeks easier. This report may also help you to identify specific tasks that can be handled by your collaborators while you are on leave (see rule #4). And for coders: always annotate your code! In the UK, you can arrange for optional isolated work days, “keep-in- touch days” to have a softer start (for example, one or two days per week for the last one or two months of your leave).

### 3. Work on your next contract

Depending on how many months you have left on your contract, you may need to plan your next move before your leave. At that point, you should take advantage of the fellowships targeted at researchers with career breaks or caring responsibilities, and those that provide extensions to applicants on maternity leave. Also, many funding agencies discount any time spent on leave for the calculation of time from the PhD required on their eligibility criteria (e.g., European Research Council). This is something that supervisors can and should be supportive of, pointing the postdocs to these opportunities and encouraging them to apply [27]. You may be able to start working on your applications, or even apply, before your leave.

For specific job positions, do not hesitate to apply; it is not unusual to request a long-delayed start that could cover your maternity leave and your remaining contract time.

### 4. Create a strong and supportive collaboration network

This can be general advice for all postdocs, but particularly important for postdoc mums. Collaborations will allow you to share leading responsibilities, delegate work, stay in touch with the community, and participate in other research projects, all crucial to supporting the career of recent mums and making them feel supported. It is a particularly good moment to reflect on your collaborative relationships and rely on those which are mutually beneficial and supportive. It may be too late to start doing this once you are already pregnant, so plan ahead!

### 5. Let your collaborators know what to expect

It is beneficial if you provide collaborators with the opportunity to take your leave into account. Giving them the chance to get from you anything they may need before you go on leave would ensure that you are kept in the loop and not excluded for not providing data on time or not approving the manuscript. Also, let your collaborators know what they can expect in terms of your availability: will you reply to urgent emails? Will you not check your email at all? Especially, chat with them about any upcoming projects that you should be involved in; do not let them exclude you just because you may not be fully available during your maternity leave. You may not be able to contribute for the first months, but usually there are plenty of opportunities to contribute at different stages of a manuscript or grant application. If nothing else, by addressing the issue widely and beforehand, you might create some peer pressure to modulate the behaviour of any potential unsupportive collaborators. An informative out-of-office automatic reply can be very helpful during your leave.

If upon your return, you learn you have been excluded, do not hesitate to reach out and express your interest in getting involved.

### 6. Be protective of your work time

It is good advice in general, but particularly in this situation. It is the perfect moment to start saying no to optional tasks like reviewing manuscripts, volunteering for events, and similar requests. Think strategically about what is a good time investment in order to get a permanent job. Be protective of your work time but also of your personal time. Learning some time-management strategies would be highly beneficial – especially before getting pregnant, when it might be easier to participate in a time-management workshop.

### 7. Contextualise your career trajectory

When you apply for jobs or fellowships, you should be able to be open about your caring responsibilities and the impact they have had on your career progression. You could use a few lines to explain why (if) your productivity has decreased in the last years or why you have not attended any conferences. Evaluation committees should appreciate the context and take it into account when making decisions, especially when working toward inclusive academic environments [27]. If you feel that the environment is not supportive and would not welcome your honesty, you can keep your personal situation to yourself, or maybe you should reconsider whether that place is a good fit for you.

Equally, do this for yourself, do not compare yourself with other researchers with no caring responsibilities (or with!). We all have different life situations, abilities, and priorities, and it is never wise to measure your worth against other people’s achievements.

### 8. Discuss your expectations with your supervisors

Your supervisors will play an important role in your career progression. Whether they adopt a supportive attitude or not, they will have a positive or negative influence on your “academic” maternity leave experience; however, their reaction is mostly out of your hands. In a supportive scenario, we suggest you discuss openly what your expectations and needs are before and after your leave.

In the months before the leave, it is important for you to get the things that you planned to get done (rule #2) done. That could require some effort from supervisors, such as having a few extra meetings or writing a last-minute reference letter, anything that would help you leave things in the right place to return to. You should also communicate to your supervisors the level of engagement you want to keep during maternity leave and, if it is your case, highlight your desire to remain in academia afterwards.

After returning to work, there is a period where you will be adapting to being back at work, and your child will be adapting to being without you (whatever the arrangement is) - and it is hard. You may have to cancel a meeting at the last-minute or accumulate a bit of a delay in a project; presenting these possibilities to your supervisors ahead of time should avoid surprises and make things easier. Similarly, discussing with them the possibility of working flexible hours, part-time, or remotely if that suits you [25] will improve your experience. It would be useful to agree on having the lab/group meetings during school/nursery hours, so it is more likely for you to be able to attend and remain engaged with the group.

In the case of an unsupportive scenario, you need to be more strategic to minimise the potential negative impact, for example, by discussing your plans and requests with multiple advisors/mentors present at the same time or with an HR representative. In this situation, rely on the other rules as much as possible and remember that not all labs are like that; do not let a bad experience decide your career.

Finally, in any case, you could share with your advisors some of the relevant literature on the effect of maternity leave on postdoctoral women's career progression and proposed recommendations (such as the current manuscript or anything cited here) to provide an informed background that increases their awareness of the situation and to fall back on if necessary.

### 9. Reassess and adjust your commitments

You have planned your leave, you have talked to your collaborators about what to expect during those months, you have explained to your supervisors your limitations and your expectations, and then, the time comes and you feel like you do not really want to open your email or you do not want to be in that panel—or the opposite! You might want to be more involved than you expected. That is ok. You are completely entitled to change your mind. The experience of becoming a mum can affect people in different ways, even differently than what they were expecting. If you feel your situation has changed, it is important to find the moment to communicate it to the relevant people. For example: “I have decided I am going to take it easy during my maternity leave, and I don’t think I will be able to get back to you on time/ attend the panel/ have that meeting. I apologise for the inconvenience”. Open communication about your decisions will facilitate understanding by your collaborators, advisors, and institutions, and allow them to adjust their expectations accordingly. This rule extends to the time beyond maternity leave, when your childcare responsibilities and time demands will change and evolve as the kids grow.

### 10. Raise your voice to promote inclusion in social and academic events

It is undeniable that the social sphere of academic spaces has an impact on our careers. If mums feel excluded from social events because they cannot stay after work to go for a drink, it would push them out. This sometimes goes beyond the social aspect, as sometimes ideas for new projects, grants, and collaborations are born during these leisure events, excluding those who systematically cannot assist. This has an easy solution: alternate drinks after work with breakfast before work, or lunch on Wednesdays, coffee and donuts on Thursdays, or even family walks on the weekend. If your lab/department/group of collaborators does not offer these alternatives, raise your voice. They might have never thought of it and welcome the suggestion. Being inclusive would only make these events more fun and attractive for everyone, and provide a broader range of life-work balance examples for younger academics and students.

Similarly, many academic events, working groups, conferences, away-days, require travelling and overnight stays, which become a challenge for researchers with childcare responsibilities. Get informed about potential support during the event, such as breastfeeding and family rooms in the venue and on-site childcare providers during the sessions. Sometimes, there is specific financial help for childcare support while attending these events. For example, at the British Ecological Society meetings, attendees can register with a discounted fee if they are attending with children. The German Centre for Integrative Biodiversity Research (iDiv) offers childcare for participants attending workshops with children. Take advantage of these opportunities and if they are not offered, email the organising committee; it may not be too late to make their event more inclusive (and if it is, they may learn something for the next time).

## Concluding remarks

Becoming a mother, or growing the family, is an incredibly exciting moment in life - and it should be cherished and embraced- but sometimes the dread of the potential impact on the academic career of postdocs turns it into a bitter-sweet stage. We believe academic careers are long-term races that should allow for a brief recess in order to combine professional and personal development. As this is not usually the case, we propose using these 10 simple rules to support postdocs facing motherhood.

In summary, we need to promote empathy and work together to help postdoctoral women remain competitive despite a break in their academic activity. This is done by planning ahead, collaborating, and embracing their new situation. We also need to provide these women with the right academic environment so they can stay and feel a sense of belonging and worth.

One final piece of advice for postdoc mums. When considering the way to balance the unstable situation of postdoctoral life with motherhood, bear in mind that young children are incredibly adaptable and resilient. Sometimes, it is the societal expectation of how children should be brought up more than our actual perception of what our children need that creates the stressful stability dilemma. Every family has to consider their own circumstances and only you know what is best for your kids!

We want to provide a brief disclosure to acknowledge that 1- we presented some examples of the impact of maternity on a women’s performance but there are personal circumstances that would make for different or even more challenging situations, such for example single or adoptive mothers, or pregnancy, birth or neonatal complications; 2- maternity policies and provisions, such as keep-in-touch days, vary between countries, institutions and even research fields, providing a better or worse experience; our rules can be applied everywhere but might include some regional and contextual bias; and 3- many men also face new parental duties during their postdoc years, which in many cases represent a challenge to the progression of their careers [16]. The advice given here could well be used in these situations, but it is especially targeted to women as they are the ones who, as a collective, become underrepresented in senior faculty positions as a consequence of maternity [28].

It is our hope that these small steps help to create awareness in the academic society, leading to the implementation of concrete solutions across multiple levels of the academic hierarchy to help retain postdoctoral women in academia and support them to flourish.
